# Online46: Online cognitive assessments in elderly cohorts—The British 1946 birth cohort case study

**DOI:** 10.1002/dad2.70098

**Published:** 2025-06-09

**Authors:** Ziyuan Cai, Valentina Giunchiglia, Rebecca Street, Martina Del Giovane, Kirsty Lu, Maria Popham, Andrew Wong, Heidi Murray‐Smith, Marcus Richards, Sebastian Crutch, Peter J. Hellyer, Jonathan M. Schott, Adam Hampshire

**Affiliations:** ^1^ Department of Brain Sciences, Imperial College London, Burlington Danes The Hammersmith Hospital London UK; ^2^ Department of Neuroimaging, Institute of Psychiatry, Psychology and Neuroscience King's College London London UK; ^3^ Department of Biomedical Informatics Harvard Medical School Boston Massachusetts USA; ^4^ Department of Neurodegenerative Disease The Dementia Research Centre, UCL Queen Square Institute of Neurology University College London London UK; ^5^ UK Dementia Research Institute Care Research and Technology Centre Imperial College London and the University of Surrey, Sir Michael Uren Hub London UK; ^6^ MRC Unit for Lifelong Health and Ageing University College London London UK

**Keywords:** birth cohort, cognitive task, compliance, elderly cohort, online assessment, participation, qualitative feedback

## Abstract

**INTRODUCTION:**

Online assessments are scalable and cost effective for detecting cognitive changes, especially in elderly cohorts with limited mobility and higher vulnerability to neurological conditions. However, determining the uptake, adherence, and usability of these assessments in older adults, who may have less experience with mobile devices, is crucial.

**METHODS:**

A total of 1776 members (aged 77) of the Medical Research Council National Survey of Health and Development (NSHD) were invited to complete 13 online cognitive tasks. Adherence was measured through task compliance, while uptake (consent, attempt, completion) was linked to health and sociodemographic factors. Usability was evaluated through qualitative feedback.

**RESULTS:**

This study's consent (56.9%), attempt (80.5%), and completion (88.8%) rates are comparable to supervised NSHD substudies. Significant predictors of uptake included education, sex, handedness, cognitive scores, weight, smoking, alcohol consumption, and disease burden.

**DISCUSSION:**

With key recommendations followed, online cognitive assessments are feasible, with good adherence and usability in older adults.

**Highlights:**

- Online cognitive tasks have good uptake, adherence, and usability in older adults.
- Education, previous cognitive scores, and alcohol consumption predict consent.
- Alcohol consumption and weight are related to attempting an assessment.
- Sex, smoking, and disease burden are associated with completion.
- Protocol challenges and recommendations are identified through qualitative analysis.

## 1 BACKGROUND

Cognitive tasks are crucial for detecting and monitoring age‐related decline. Traditionally, these assessments are conducted in pen‐and‐paper format under supervised conditions, tailored to specific cognitive domains and clinical populations.^1^ Automated online assessments offer an alternative approach with several advantages. They can match or surpass popular pen‐and‐paper tasks in detecting cognitive impairments,^2^, ^3^, ^4^, ^5^ reduce the burden on patients and clinical staff because patients can conduct assessments in unsupervised environments,^6^ and enable cost‐ and time‐effective large‐scale longitudinal data collection.

Online assessments are particularly beneficial for older adults, who may struggle to attend clinics in person. However, technology anxiety and limited access to devices may pose challenges to engagement,^7^, ^8^ making determining the uptake of online testing technology in this age range essential. Additionally, unsupervised cognitive tasks may introduce data errors due to the lack of support, necessitating robust investigation of adherence, usability, and sampling bias.

Previous studies have assessed the validity of online assessments both by measuring their ability to discriminate clinical conditions^9^, ^10^, ^11^ and by comparing their performance to standard pen‐and‐paper tasks^12^ or supervised digital tasks.^13^ However, they typically lack detailed analysis of factors influencing adherence to cognitive assessments or are not focused on aging populations.^14^ Here, we examine the application of computerized cognitive assessments in an elderly cohort (age = 77) from the Medical Research Council (MRC) National Survey of Health and Development (NSHD). Our first aim was to assess uptake when recruiting an older adult cohort to a 40‐minute online assessment and to determine the factors that predict the probability of participation and completion. Our second aim was to assess patterns of adherence and compliance. Finally, we aimed to qualitatively analyze information on usability and barriers for participation and to provide recommendations for future protocol optimization.

## 2 METHODS

### 2.1 The 1946 British birth cohort study sample

The MRC NSHD is the longest continually studied British birth cohort, comprising 5362 individuals born in England, Scotland, and Wales during the first week of March 1946.^15^ The study has collected sociodemographic, medical, cognitive, and psychological function data through interviews and examinations in 27 waves and smaller substudy collections with ≈ 2700 participants in active follow‐up.^16^ The current aim of the NSHD study is to explore long‐term aging and how it is affected by factors across the life course.^17^, ^18^, ^19^, ^20^

### 2.2 Study recruitment

**FIGURE 1:**
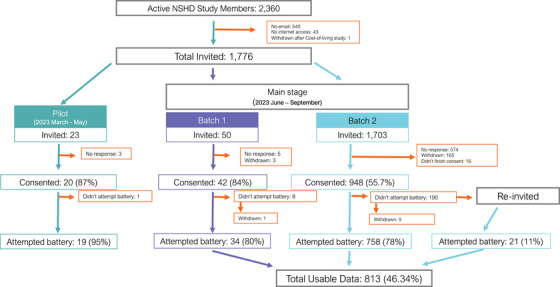
Recruitment flowchart for the Online46 study. Out of 2360 active NSHD members, 1776 were invited to participate in the Online46 study. A total of 1010 members consented and 813 of them attempted the online cognitive battery. The study involved two stages: (1) a pilot stage between March and May for assessment design and (2) a main stage between June and September for data collection. Study members from the NSHD advisory panel were selected to participate in the pilot stage (*N* = 23). The main stage was completed in two parts, a soft launch (batch 1), a small actively engaged subset of participants, who previously participated in Insight46,^18^ the neuroimaging subsample of NSHD (*N* = 50) and a full launch (batch 2), a week later with the remaining NSHD participants. NHSD, National Survey of Health and Development.

This study assessed the uptake, adherence, and usability of automated online assessments in the NSHD cohort in 2023. It involved a pilot stage to design the assessments and a main stage for large‐scale data collection. Twenty‐three study members from the participant advisory panel piloted the study. The main stage had a soft launch (batch 1) with a small actively engaged subset of participants (*N* = 50), and then a full launch (batch 2) a week later with the remaining (*N* = 1703) participants (Figure 1). Participants had 4 weeks to complete the online assessments. Follow‐up e‐mail invitations were sent after 2 and 3.5 weeks.

We recruited from all active NSHD study members (*N* = 2360), excluding those with no e‐mail (*N* = 540), no internet access (*N* = 43), or who withdrew in previous data collection (*N* = 1). A total of 1776 members were invited through e‐mails. Participants completed consent forms via a Qualtrics link and received a user‐specific link to the cognitive assessment. After 4 weeks, non‐attempting participants (*N* = 190) were re‐invited and were given an extra week to attempt the battery.

### 2.3 Cognitive task battery

Cognitron is a server system used for online cognitive assessments. It hosts > 100 optimized cognitive tasks that are sensitive, domain specific, and widely validated in the general population and clinical cohort.^2^, ^9^, ^10^, ^21^, ^22^, ^23^, ^24^ More information is available on the website: https://www.cognitron.co.uk/.

**RESEARCH IN CONTEXT**

1. **Systematic review**: A PubMed search showed that, while the validity of online cognitive assessments has been evaluated across various cohorts, quantitative and qualitative analysis on factors affecting participation in online assessments, particularly among older adults, is lacking.
2. **Interpretation**: The participation rate of elderly cohorts in the online assessment was comparable to face‐to‐face studies. Once started, completion rates for older adults were very high, with the largest dropout being at recruitment. Higher education, cognitive scores, and moderate alcohol consumption were associated with higher consent rates. Females and healthier participants were more likely to attempt and complete the battery. Qualitative analysis identified pragmatic recommendations for online assessments in elderly cohorts including enhancing instruction visibility and audibility and providing performance result summaries.
3. **Future directions**: Future research should explore: (1) methods to increase engagement at recruitment and (2) retention and attrition rates with repeated online assessments.

Here, participants were asked to undertake 13 Cognitron tasks selected to measure different aspects of reaction time, motor control, memory, attention, reasoning, and executive functions (Figure S1, Table S1 in supporting information). Participants could access the tasks through web browsers on any smartphone, tablet, or personal computer. The tasks were presented as a battery in fixed order without supervision. General instructions were provided at the beginning of the battery. Specific instructions were presented before each task followed by a brief set of practice trials. Information related to performance (accuracy and reaction time), compliance (e.g., time spent outside of the task webpage), and detailed trial‐by‐trial responses was collected automatically.

### 2.4 Uptake

We investigated how sociodemographic and health‐related characteristics differed at three levels of recruitment: (1) consented to take part, (2) attempted any of the tasks, and (3) completed the entire online assessment. The sociodemographic and health‐related characteristics of the NSHD cohort have been described previously^19^ and are summarized in Table S2 in supporting information. To evaluate the representativeness of the sample, chi‐square tests were conducted to compare the distribution of demographic categories between participants who attempted the battery (*N* = 813) and the rest of the cohort (*N* = 963).

Three multivariable logistic regression models were conducted to predict consent, battery attempt, and completion, using the same independent variables: sociodemographic factors, mental health, smoking history, alcohol consumption, body mass index, disease burden, and self‐rated health. Statistical significance was assessed using a Wald test after fitting the models. Multivariable regression was used to align with previous NSHD recruitment studies^19^ and control for confounding variables. Whether the accuracy score on the first task (Object Memory Immediate) predicted the likelihood of completing the entire assessment was evaluated with a logistic regression model.

### 2.5 Adherence

#### 2.5.1 Task compliance

Based on the task design, different measures of compliance were assessed. Conservative criteria were applied to prevent the removal of clinically relevant data. For Choice Reaction Time (CRT) and Word Definitions, the number of time‐out trials, defined as trials in which participants do not interact with the stimulus, was calculated. Participants with at least 90% of trials being time‐out trials were marked as not complying. For Verbal Reasoning, Manipulation 2D, Switching Stroop, CRT, and Object Immediate and Delay, instances of clicking repetitively in the same screen location were measured. Participants who pressed in the same location in all trials of a given task were defined as not engaged. For all tasks, participants who missed the stimulus in all trials were considered unengaged. Finally, participants with summary scores below the expected threshold—a sign of not completing the tests properly—were flagged for probable non‐compliance. These thresholds were determined from normative data (Great British Test data) with an automatic algorithm.^24^, ^25^ Specifically, the method processes the histogram distributions and compares the bin heights against predefined thresholds (i.e., the 5th percentile of the left tail of the distribution). Details can be found in Supporting Information S1.

#### 2.5.2 Cheating

Digit Span requires participants to memorize a sequence of digits that increases by one digit at each trial. A small proportion of participants write the digits in another browser window to move forward in the assessment. Participants within the top 10% quantile of the Focus Loss time distribution (i.e., time that participants spend clicking outside the task browser page during the assessment) and within the top 10% quantile of the accuracy scores were flagged as possibly cheating.

### 2.6 Usability

Telephone and e‐mail helplines were given to support participants with any technical difficulties. We captured unprompted feedback in a detailed log. Qualitative data comprising correspondence between NSHD personnel and study members were examined using thematic analysis (TA) to provide insights into the study members’ experiences.^26^

Correspondences were anonymized and compiled into NVivo,^27^, ^28^ a qualitative and mixed methods research software. The e‐mail conversation transcripts consisted of 200 e‐mails (9475 words). Detailed notes were taken by the NSHD personnel when responding to telephone queries, consisting of 78 phone calls (2306 words). Themes from the data were generated using the revised steps suggested by Braun and Clarke.^26^ All steps were completed by one rater. The same participants could contact the helpline with multiple queries, which could result in several thematic codes.

## 3 RESULTS

### 3.1 Sample features

Out of the 1776 members contacted, 1010 (56.9%) consented to participate, and 813 consented individuals attempted the battery (80.5%). Participants who attempted the battery had an almost balanced female‐to‐male ratio (52.2% female), with their highest education predominantly at A‐level (36.2%) and were mostly right‐handed (92.4%). In addition, because they were a British birth cohort, they were all 77‐year‐old White participants with English as a first language. Distributions of demographic factors, socioeconomic status, and health‐related features are presented in Table S3 and Figures S2‐S4 in supporting information. Notably, chi‐square tests show that, apart from sex, handedness, mental health, and overall disease burden, the demographic distribution differs significantly between participants and the rest of the invited cohort (Table S4 in supporting information).

### 3.2 Adherence

#### 3.2.1 Completion rate for the battery and tasks

**FIGURE 2:**
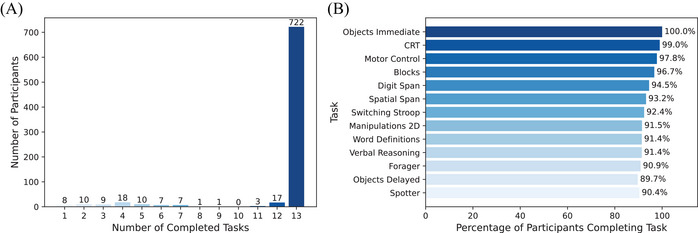
Completion rate for the battery and tasks. (A) Cognitron completion rate, indicating the number of participants with various counts of completed tasks. (B) Task completion rate, with tasks arranged in order from top to bottom according to the battery sequence.

Among the 813 participants who attempted the battery, 722 (88.8%) completed all 13 tasks in the assessment (Figure 2A). The average completion time was 40.68 ± 4.24 minutes (Table S5 in supporting information). Most participants with 12 tasks completed all but the Objects Delayed (*N* = 10), which was expected because it is not administered if not completed within a fixed timeframe on the same day as the Objects Immediate task. More details on the completion rate are in Figure 2B. As expected, Objects Immediate (the first task) had a 100% completion rate. The last task (Spotter) had a completion rate of 90.4%.

#### 3.2.2 Most tasks were characterized by a 100% compliance rate

The task compliance and cheating analysis results are reported in Figure S5 and Table S6 in supporting information. Of the 13 tasks studied, 8 had a compliance rate of 100%. In the other four tasks (Spatial Span, Digit Span, 2D Manipulations, and Spotter), < 2% of participants showed signs of probable non‐compliance. The most common sign of probable non‐compliance in these tasks consisted of participants having an accuracy score lower than the expected threshold (e.g., failing the simplest trials).

Specifically, CRT was characterized by 9% (*N* = 67) of participants who had issues such as not recording > 90% of the trials (*N* = 56) or clicking repetitively in the same screen location (*N* = 19). Detailed numbers are shown in Table S6 in supporting information. While the CRT lacks metrics (e.g., “focus loss”) that distinguish slowed processing from intentional disengagement, it is likely that these issues are related to task design problems rather than participant compliance, noting that older participants, who often require longer to respond, are more likely to exceed the 1000 ms response limit of trials on this task.

### 3.3 Uptake

#### 3.3.1 Higher education, greater adult verbal memory, and regular alcohol consumption correlate with an increased likelihood of study consent

**FIGURE 3:**
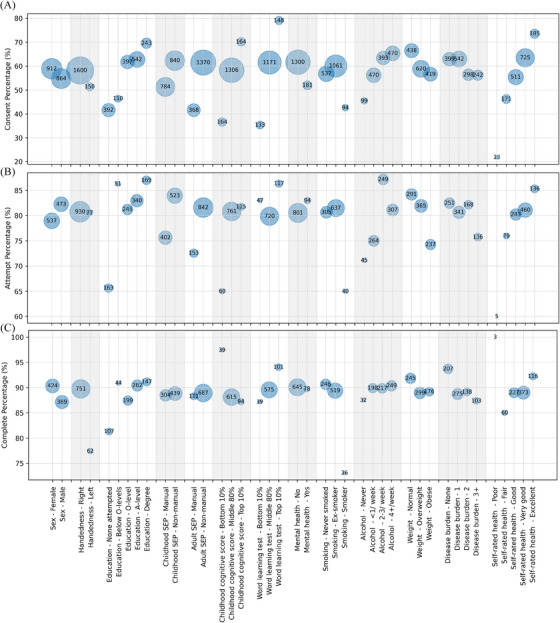
Relationship between uptake of online cognitive tasks and sociodemographic and health‐related features. The size of each circle is proportional to the number of participants in the corresponding category, with numbers also displayed within the circles. The position of the circles represents the percentage of participants in each category who consented, attempted or completed the online battery in panels A, B and C, respectively.

Consent rates for the pilot, batch 1, and batch 2 of the study were 87%, 84%, and 55.7%, respectively (Figure 1). The multivariable logistic regression model was significant in predicting study consent (pseudo *R* ^2 ^= 0.09, *N* = 1137, log‐likelihood ratio [LLR] *p* < 0.001). Pseudo *R* ^2^ is a goodness‐of‐fit measure that indicates the improvement in model likelihood over the null model, with higher values representing better model fit. According to the Wald test, education level (*χ* ^2^ = 9.70, *p* = 0.046), word list learning test at age 69 (*χ* ^2^ = 11.8, *p* = 0.003), and alcohol consumption (*χ* ^2^ = 8.6, *p* = 0.035) were significant predictors. Specifically, compared to members without educational attainment, individuals with a degree were more likely to consent (*p* = 0.01). Regarding the word list learning test, those who scored in the top 10% (*p* = 0.001) and 80% middle were more likely to consent to the study (*p* = 0.003) than those with the lowest 10% score. Finally, members with a higher level of alcohol consumption were more likely to consent to the study (“four or more times a week,” *p* = 0.012, and “two or three times per week,” *p* = 0.047; Table 1, Figure 3A). The full model outputs are detailed in Table S7 in supporting information.

**TABLE 1:** Logistic regression models on demographic variables for battery consent, attempt, and completion.

	Consent			Attempt			Complete		
Variable	OR	*p*	Wald test *p*	OR	*p*	Wald test *p*	OR	*p*	Wald test *p*
Sex									
Female	Reference								
Male	0.79	0.11		1.04	0.84		0.51	**0.03**	
Handedness									
Right	Reference								
Left	0.68	0.11		0.94	0.88		0.39	**0.04**	
Education			**0.046**			0.06			0.16
Non‐attempted	Reference								
Below O‐levels	0.89	0.69		4.22	0.01		1.75	0.39	
O‐level	1.40	0.10		1.51	0.18		1.50	0.37	
A‐level	1.43	0.07		1.84	0.04		2.58	0.04	
Degree	2.10	**0.01**		2.12	0.05		3.55	0.03	
Word learning test memory score at age 69			**0.003**			0.52			0.68
Bottom 10%	Reference								
Middle 80%	2.86	**0.003**		0.59	0.28		1.56	0.10	
Top 10%	1.98	**0.001**		0.68	0.51		1.00	0.58	
Lifetime smoking by 69 years			0.08			0.26			**0.048**
Never smoke	Reference								
Ex‐smoker	1.18	0.26		1.16	0.24		1.04	0.91	
Current smoker	0.63	0.14		0.57	0.50		0.19	**0.02**	
Alcohol use at age 69			**0.04**			**0.02**			0.72
Never	Reference								
< 1 per week	1.40	0.23		1.73	0.2		1.53	0.53	
2–3 per week	1.75	**0.05**		3.42	**0.01**		1.49	0.56	
4+ per week	2.02	**0.01**		1.70	0.21		1.96	0.32	
Weight status at age 69			0.45			**0.04**			0.99
Normal	Reference								
Overweight	0.82	0.21		1.01	0.98		0.99	0.97	
Obese	0.87	0.46		0.57	**0.04**		0.99	0.97	
Overall disease burden at age 69			0.59			0.58			**0.04**
0	Reference								
1	1.24	0.22		0.79	0.37		0.30	**0.01**	
2	1.03	0.88		1.00	1.00		0.37	**0.04**	
3 and 3+	1.67	0.77		0.69	0.27		0.25	**0.01**	

*Note*: This table presents the significant findings from three separate multivariable logistic regression models predicting (1) study consent, (2) battery attempt, and (3) battery completion, based on the same set of demographic and health‐related independent variables. Consent: Number of observations = 1137, Pseudo *R*‐squared = 0.09, LLR *p* < 0.001. Attempt: Number of observations = 724, Pseudo *R*‐squared = 0.08, LLR *p* < 0.05. Completion: Number of observations = 585, Pseudo *R*‐squared = 0.10, LLR *p* = 0.04. ORs and *P* values are shown for variables that reached significance in at least one of the three models. For each demographic variable, significant Wald test *P* values are shown in bold. For those variables with significant Wald test results, significant *P* values from individual categories in the logistic regression model are also shown in bold. Wald test results are not reported for variables with only two categories (e.g., sex), as their significance is directly assessed in the regression model. Full model outputs (including non‐significant variables) are provided in Table S7 in supporting information.

Abbreviations: LLR, log‐likelihood ratio; OR, odds ratio.

#### 3.3.2 Participants with moderate alcohol consumption and healthier weight are more likely to attempt the battery

The multivariable logistic regression model that predicts battery attempt was significant (pseudo *R* ^2^ = 0.08, *N* = 724, LLR *p* < 0.05). The Wald test identified alcohol consumption (*χ* ^2^ = 10.3, *p* = 0.02) and weight (*χ* ^2^ = 6.3, *P *= 0.04) as significant predictors. Members with a higher level of alcohol consumption were more likely to attempt the battery (“two or three times per week” *P* = 0.01), compared to non‐drinkers. Regarding weight, obese members were less likely to attempt the battery (*p* = 0.04; Table 1, Figure 3B).

#### 3.3.3 Battery completion is less likely among male, left‐handed, current smokers, and individuals with higher disease burden and lower performance in the first task

The multivariable logistic regression model predicting battery completion was significant (pseudo *R* ^2^ = 0.10, *N* = 585, LLR *p* = 0.04). The Wald test identified four significant predictors of battery completion: sex (*χ* ^2^ = 4.67, *p* = 0.03), handedness (*χ* ^2^ = 4.44, *p* = 0.04), smoking by age 69 (*χ* ^2^ = 6.07, *p* = 0.048), and disease burden at age 69 (*χ* ^2^ = 8.32, *p* = 0.04). Specifically, male participants (*p* = 0.03), left‐handed participants (*p* = 0.04), and current smokers (*p* = 0.02) were less likely to complete the battery. Additionally, an increased disease burden was associated with a lower likelihood of completing the battery (disease burden of 1, 2, 3+, has *P* values of 0.01, 0.04, and 0.01, respectively). The rates of completing the whole battery for participants with no disease burden, and those with 1, 2, and 3+ disease burdens were 93.7%, 88.3%, 89.1%, and 86.4%, respectively (Figure 3C). Due to the low number of observations, a sensitivity analysis was conducted for battery completion. Specifically, univariate regression models were fitted for individual variables, resulting in odds ratios within a similar range (Table S8 in supporting information).

Furthermore, performance in the first cognitive task was also a significant predictor of battery completion (*N* = 813, LLR *p* = 0.03), with higher summary scores correlating with a greater likelihood of completion (*p* = 0.03; Table S9 in supporting information).

### 3.4 Usability

#### 3.4.1 TA of qualitative feedback

**FIGURE 4:**
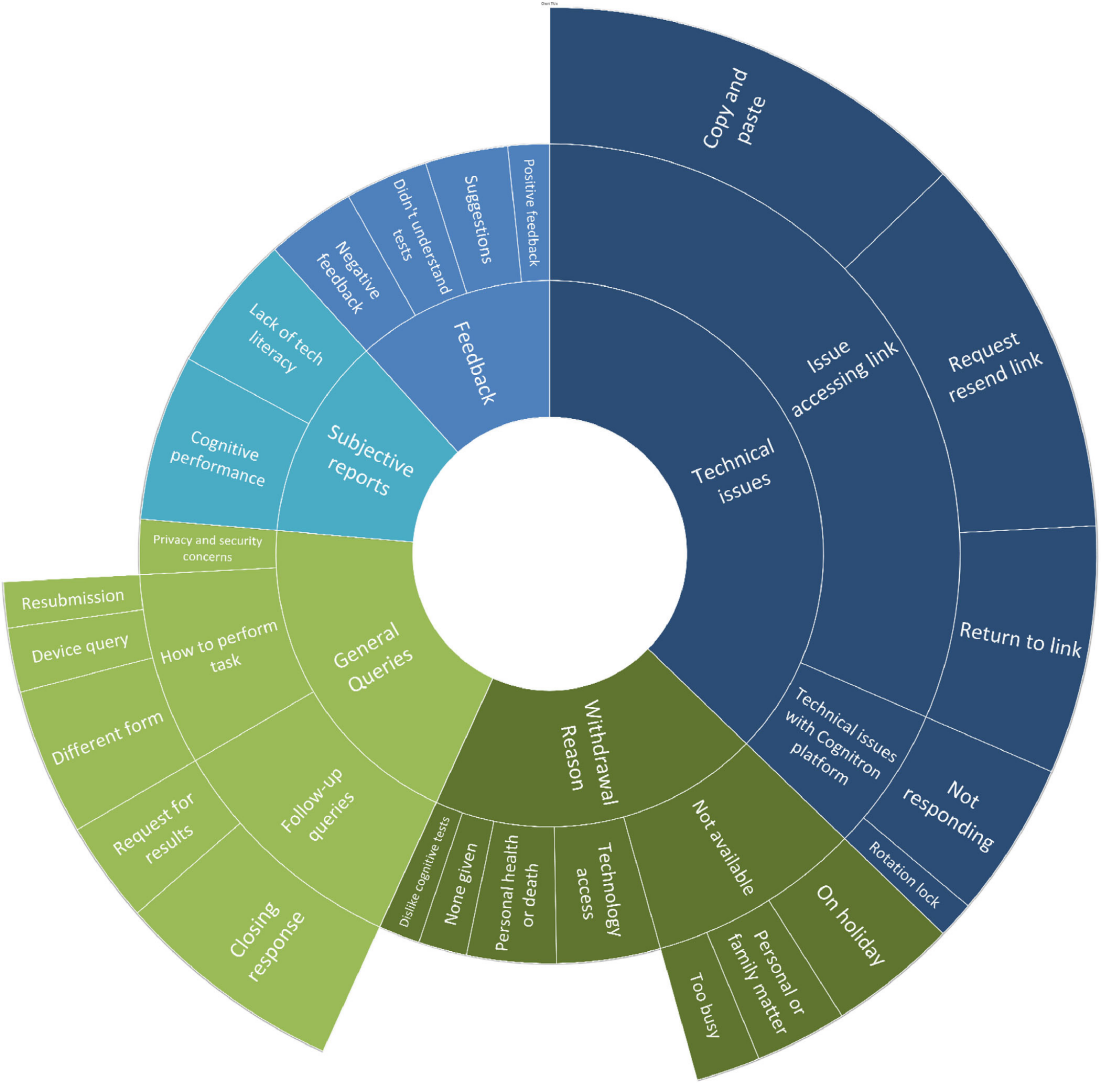
Qualitative feedback for online cognitive tasks. The sunburst diagram represents the results of the qualitative analysis, showing the proportion of data for each theme (inner circle) and subtheme.

The inductive TA^29^ identified five key themes (Figure 4, Table S10 in supporting information) summarizing the reasons participants contacted the helpline: technical issues (*N* = 137), general queries (*N* = 72), reasons for withdrawing (*N* = 72), subjective reports (*N* = 44), and providing feedback (*N* = 43).

#### 3.4.2 Technical issues

The technical issues were divided into two subthemes: issues using the link (*N *= 116) and issues with the platform (*N* = 21). Participants sometimes could not navigate between the Qualtrics consent website and the Cognitron platform. After consenting, participants received their user‐specific link and were instructed to copy and paste it into another window or click the e‐mailed link. This subtheme could be further divided into three issues: unable to “copy and paste” (*N* = 47), “requests to resend link” (*N* = 42), and unable to “return to link” (*N* = 27) after an hour of inactivity. Fewer participants contacted the helpline for difficulties with the Cognitron website. Enquiries were categorized into two subthemes: “Cognitron website not responding” (*N* = 17) and “issues with rotation lock on tablet device” (*N* = 4). The webpage did not respond due to poor internet connection or outdated web browsers. The rotation lock occurred on tablets as the platform requests portrait screen orientation.

#### 3.4.3 General queries

General queries had three subthemes: follow‐up queries (*N* = 36), how to perform the task (*N* = 28), and privacy and security concerns (*N* = 8). These subthemes included participants who made a “request for results” (*N* = 11), while others provided a closing response (*N* = 25; e.g., “I have now completed the tasks.”). Participants who asked “how to perform the task” either had a “device query” (*N* = 7), asked if they could complete the tasks in a “different form” (*N* = 16) or if they could go back to the tasks later—“resubmission” (*N* = 5). Some participants (*N* = 8) had privacy and security concerns (e.g., “What happens between it [the data] being sent and received?”).

#### 3.4.4 Reasons for withdrawing from Online46

The withdrawing reasons were dislike of cognitive tasks (*N* = 6), none given (*N* = 7), not available (*N* = 31), personal health or death (*N* = 13), and technology access (*N* = 15). Study members were not available for three reasons: on holiday (*N* = 14), personal or family matters (*N* = 10), and too busy (*N* = 7).

#### 3.4.5 Subjective reports

The subjective reports provided insights into participants’ perceptions of online cognitive testing and can be divided into two subthemes: lack of technical literacy (*N* = 20) and cognitive performance (*N* = 24). The lack of technical literacy subtheme highlights the participants’ perception of “modern on‐line technology.” Eight participants also provided commentary on their perceived cognitive performance, highlighting the participants’ negative perception of their cognition.

#### 3.4.6 Providing feedback

The helpline received negative (*N* = 13) and positive feedback (*N* = 6), suggestions (*N* = 12), and comments from those who did not understand the tasks (*N* = 12).

## 4 DISCUSSION

We completed a quantitative and qualitative analysis of the uptake, adherence, and usability of unsupervised computerized cognitive assessments in an elderly general population birth cohort, with the goal of informing future studies and leading the application of such assessments in clinical research and practice.

We confirmed that the participation rate of elderly cohorts at different stages of recruitment is adequate. Specifically, the overall consent rate of 56.9%, of whom 80.5% attempted and 88.8% completed the battery, was comparable to previous substudies conducted under supervised conditions.^19^ At just above 55%, the consent rate was similar to Insight 46 (a substudy of the NSHD), where out of the 1322 eligible participants, 59% were willing to attend a clinic visit in London, and of the 841 members invited, 60% were recruited and attended a research visit.^19^

The duration of the assessment (40.68 ± 4.24 minutes) was within the range of other self‐administered online cognitive assessments, such as the Touch panel‐type dementia assessment scale (30 minutes)^30^ and short‐form MicroCog (30–45 minutes). The qualitative feedback did not indicate any complaints for the duration of the assessment;^31^, ^32^ therefore, a 40‐minute cognitive assessment may be well received by elderly cohorts, although shorter batteries are more appropriate for repeat testing over short periods.

Our study showed that consent, participation, and completion of the online assessment related to sociodemographic and health‐related factors. Higher education increases the likelihood of consenting, likely due to greater willingness to learn and use technology,^33^ improved understanding of research information,^34^ and increased confidence in performance.^35^, ^36^ This aligns with previous findings from NSHD substudies,^19^, ^35^ other web‐based cognitive batteries,^37^ and epidemiological studies.^38^, ^39^, ^40^ Similarly, participants with higher past cognitive scores were more likely to consent, possibly due to greater overall health literacy.^35^, ^41^, ^42^ Consistently, participants from the pilot and batch 1 of the study recruitment stage had higher consent rates than those in batch 2, as expected. Batch 1 participants had volunteered to take part in Insight46 (an involved, in‐person substudy) and had better education and self‐rated health.^19^ Clinicians and researchers could use more targeted recruitment strategies to reach populations with lower health literacy, thereby improving consent rates and broadening representativeness. Additionally, childhood cognitive scores can be adjusted in the downstream analysis of the Online46 study.^43^

Interestingly, participants who consume alcohol more than twice a week were more likely to consent and attempt the battery than non‐drinkers. Previous studies link moderate alcohol consumption with better education, higher socioeconomic status,^44^, ^45^ and better psychological and physical well‐being,^46^, ^47^ which could make regular and moderate drinkers more proactive in health assessments. Supporting this hypothesis, participants who completed all tasks or had higher completion rates were less likely to be current smokers, obese, or have a high disease burden. This suggests that individuals with better health are more likely to complete the battery.

Overall, these biases should be considered when interpreting results on this cohort, as the strength of associations might not be representative of the whole population. However, this bias might not be problematic in studies assessing the relationship between health and exposures across the life course.^48^ This was also true for previous NSHD studies.^16^, ^19^

The qualitative feedback allowed us to identify recommendations for improving accessibility in online cognitive assessment of elderly cohorts (Table 2). First, although compliance rates for individual tasks were high, streamlining the process from recruitment through to starting the battery is important to prevent challenges and dropout when moving between platforms and task stages. Because Cognitron was deployed remotely, older adults may have faced additional barriers such as limited digital literacy. However, it is designed for both online and supervised deployment; therefore, future studies could offer supervised or partially supervised options—for example, inviting participants by SMS if they lack e‐mail, or posting to a clinic or visiting them at home to help complete the digital assessment, which has been successfully implemented previously.^2^, ^5^, ^49^, ^50^ Clear, concise written or video instructions should be provided before each stage of the battery, and the inclusion of practice trials is advisable. Large fonts and/or audio instructions are important for elderly cohorts and user‐friendly interfaces with clear language and intuitive icons. Moreover, task difficulty levels could be tailored (e.g., lengthening response time windows or adjusting stimulus complexity) to capture more granular variability in older adults’ cognitive and sensory abilities. The qualitative feedback shows that participants vary in their desire to view their performance reports. A simple interpretable summary should be provided at the end but shown only to participants who opt to see it. Then, a detailed Q&A should be available with task instructions and a summary of privacy and data‐sharing regulations. This should complement an active helpline to address any potential engagement and technical issues. Finally, most elderly participants who begin an online assessment tend to finish it—the greatest bias occurs at the point of recruitment into the study. Further investigation is warranted into how to increase engagement at the recruitment stage.

**TABLE 2:** List of challenges and recommendations identified through the qualitative analysis.

**Challenges**	**Recommendations**
Stressful tests	Opportunity to complete the assessment across multiple days, with possibility to easily re‐access the platform where the test is hosted and system of reminders
Issues accessing the tests online	1. Detailed video/written instructions to guide participants on how to access the assessment 2. Simplification of the process by having the consent and assessment within the same platform
Feedback and results from online assessment	1. Design of automatic results’ reports with summary of cognitive abilities 2. Possibility for users to decide whether to see the results or not, to prevent negative feelings toward their cognitive abilities 3. Possibility for users to obtain either a summary of performance or a measure of comparison toward other participants
Issues with test website freezing or not responding	1. Reminder to ensure testing environment has stable internet connection 2. Availability of phone and/or e‐mail helpline to handle potential issues with the assessment platform
Older adults physical condition interrupts or hinders the completion of assessment	1. Design assessments to allow pausing between tests and restarting it on the same day (or another day) 2. Design of the interface such that it is easier for older cohorts (e.g., bigger font sizes, possibility to hear written instructions, limited flashing lights …)
Lack of technical literacy	1. Design of simplified interface with clear language and intuitive icons 2. Simplification of the consent and assessment process 3. Add an option for participants to run the tests with supervision.
Uncertainty for how to conduct the test	1. Detailed Q&A webpage 2. Task‐specific instructions before each task 3. Practice trials before the tests 4. Interactive tutorials that walk participants through the practice sessions
Unavailability to complete the test	1. Possibility to complete the assessment throughout a longer timespan 2. Multiple follow‐up e‐mails
Privacy and authenticity concerns	1. Use of previously known e‐mail domain when contacting participants 2. Advertisement of study in e‐mail and/or paper newsletters 3. Easily accessible summary of privacy and data‐sharing regulations (e.g., inclusion in the Q&A webpage) that is not embedded in extensive text

Our study has some limitations. First, we could not evaluate variability by age in this birth cohort. All participants are White, and while they represent the population in post‐war Britain, they may not be representative of today's population diversity.^35^ Additionally, the demographics of participants did not broadly represent the whole cohort, and they may have greater motivation to engage in the study. Nevertheless, the study focused on quantifying factors that underlie self‐selection bias. As a longitudinal study, NSHD still offers valuable insights into how exposures relate to health outcomes over time, which does not strictly require broad representativeness.^51^ The small size of some subgroups is another limitation that affects the reliability of evaluation; for example, those who were left handed showed lower completion rates here, but such bias has not been evident in larger population studies using the same technology.^22^ Additionally, out of the 2360 active NSHD members, 583 individuals did not have e‐mail or internet access and were not included in the uptake analysis, an important selection bias. The definition of probable non‐compliance (i.e., below expected performance thresholds) may label participants with cognitive dysfunction into the probable non‐compliance group. Feedback was solely collected from participants with issues while conducting the assessment, which tends to highlight negative aspects of online assessments and introduce selection bias; the high completion rates support that the assessment was generally well received. Information about participants’ retention and attrition rates with repeat online assessment will only be available after future data collection. Further research will explore the association between online cognitive assessment results and biomarkers, with findings to be published separately. Given the adequate participation rate and the demographic predictors identified for an elderly cohort, this online cognitive battery is valuable for both neuroscience studies and broad applicability in fields such as demography, economics, and education research. In conclusion, online cognitive assessments are feasible for elderly cohorts if design considerations pertaining to accessibility, engagement, and compliance are followed.

## CONFLICT OF INTEREST STATEMENT

Z.C. is pursuing a PhD cofunded by H2 Cognitive Designs Ltd, which produces online assessment technology and provides online survey data collection for third parties. A.H. is the owner/director of H2 Cognitive Designs Ltd. and Future Cognition Ltd. P.H. is the founder and director of H2 Cognitive Designs Ltd., which develops and markets online cognitive tests. J.M.S. is the Chief Medical Officer of Alzheimer's Research UK and a clinical advisor at the UK Dementia Research Institute. He reports grants from the NIHR, LifeArc Foundation, and the British Heart Foundation. The other authors have nothing to disclose. Author disclosures are available in the supporting information.

## ETHICS STATEMENT

The study was approved by the National Research Ethics Service Committee London (REC reference 14/LO/1173), and all participants provided written informed consent.

## Supporting information



Supporting information

Supporting information
